# Risk factors and long-term outcomes of infantile colic: A nationwide population-based study

**DOI:** 10.1038/s41598-025-34646-4

**Published:** 2026-01-13

**Authors:** Fang Min Liao, Yin-Ting Chen, Shang-Po Shen, Chau-Ren Jung, An-Chyi Chen, Shu-Fen Wu, Yu-Chia Chang, Hung-Chih Lin

**Affiliations:** 1 Department of Pediatric Gastroenterology, Hepatology & Nutrition, China Medical University Children’s Hospital, China Medical University, Taichung, 40447 Taiwan; 2 Department of Public Health, China Medical University, Taichung, Taiwan; 3 Department of Neonatology, China Medical University Children’s Hospital, China Medical University, No. 2, Yude Road, North District, Taichung, 40447 Taiwan; 4 School of Medicine, College of Medicine, China Medical University, Taichung, 40447 Taiwan; 5 Japan Environment and Children’s Study Programme Office, National Institute for Environmental Studies, Tsukuba, Japan; 6 Department of Long Term Care, College of Health and Nursing, National Quemoy University, Kinmen, 892009 Taiwan; 7 Department of Healthcare Administration, College of Medical and Health Science, Asia University, Taichung, 41354 Taiwan; 8 Asia University Hospital, Asia University, Taichung, Taiwan

**Keywords:** Infantile colic, Disorders of gut-grain interaction, Allergic disorders, Neurodevelopmental disorders, Diseases, Gastroenterology, Health care, Medical research, Risk factors

## Abstract

Infantile colic is a common condition during early infancy with unclear etiology, likely involving multifactorial mechanisms and gut dysbiosis. This nationwide population-based study investigated perinatal risk factors and long-term health outcomes associated with infantile colic. Using Taiwan’s national birth cohort data from 2010 to 2015, we identified 19,191 infants with colic and 95,955 matched controls. The inclusion criteria for the infantile colic group were having two or more related ICD codes (ICD-9-CM 789.0x and 780.9) between 1 and 5 months of age. Conditional logistic regression was applied to identify associated risk factors, and Cox proportional hazard models assessed outcomes over a 5-year follow-up. Infantile colic was significantly associated with neonatal antibiotic use, cesarean delivery, maternal atopic diseases, maternal major depressive disorder, and maternal irritable bowel syndrome. Children with a history of colic exhibited higher incidence rates of disorders of gut-brain interaction, including constipation, diarrhea, irritable bowel syndrome, and other functional abdominal pain disorders. Additionally, increased risks of attention deficit hyperactivity disorder and atopic diseases were observed. These findings suggest that infantile colic may reflect early-life biological vulnerabilities linked to gastrointestinal, atopic, and neurodevelopmental outcomes.

**Supplementary Information:**

The online version contains supplementary material available at 10.1038/s41598-025-34646-4.

## Introduction

Infantile colic is a common behavior syndrome during infancy and affects 1.9% to 19.2% of infants, depending on the diagnostic criteria used^1^. Its most prominent feature is prolonged and irritable crying, that typically peaks at six weeks and disappears by approximately three to four months of age. The previous Rome III report included a version of the Wessel et al’s “rule of threes” criteria, which states that colic crying had to start and stop suddenly and occur for three or more hours/day for at least three days in a week^2^. The current diagnostic rule for infantile colic is based on the Rome IV criteria, which are simplified to include infants less than five months of age experiencing recurrent and prolonged periods of infant crying, fussing, or irritability reported by caregivers. These episodes occur without an obvious cause and cannot be prevented or resolved by caregivers^3^.

The exact etiology of infantile colic remains unclear, but it is likely multifactorial, including dysregulation of the gut-brain axis, visceral hypersensitivity, alterations in the gut microbiota, neurodevelopmental factors, and parental psychosocial issues^4–6^. Dysbiosis is thought to be an important factor in the development of infantile colic^7,8^. Subsequent episodes after dysbiosis may lead to increased gut permeability and facilitate increased low-grade mucosal, gut, and systemic inflammation^9,10^. The gut microbiota also plays a crucial role in the development and regulation of the host’s immune system. Disruptions in this balance, such as those caused by dysbiosis, can lead to abnormal immune responses and potentially result in allergic diseases^11,12^. Generally, the management of infantile colic include probiotic use (*Lactobacillus reuteri* DSM17938)^13–16^, dietary intervention, improved parental responsiveness and focused parent counselling^17–21^.

Recent studies have indicated infantile colic was associated with several other diseases, such as disorders of gut-brain interaction (DGBI), allergic disorders, and neurodevelopmental disorders^22–27^. However, no single large cohort or population-based study has investigated the multiple long-term outcomes of infantile colic comprehensively. We hypothesize that infantile colic may either represent an early manifestation of certain disorders or share a similar pathophysiological basis with these conditions. Investigating this hypothesis could provide strategies for early intervention, potentially preventing the development of related future diseases.

The aim of our study is to clarify the risk factors associated with infantile colic, including both maternal and infant factors, and to analyze the long-term health implications for infants.

## Methods

### Data sources

The data were sourced from a nationwide and population-based birth cohort from 2010 to 2015, the Taiwan Maternal and Child Health Database (TMCHD), which is maintained and managed by the Health and Welfare Data Science Center, Ministry of Health and Welfare Taiwan (HWDC, MOHW). The TMCHD contains the de-identified identity numbers of newborns and their parents, which can be linked to the Birth Certificate Database (BCD) and the National Health Insurance Research Database (NHIRD). The BCD has the perinatal information of the newborns and maternal complications, including gestational age and birth weight, covering the period from 2010 to 2015. The NHIRD in this study covers 2010 to 2020 and contains details of beneficiaries enrolled in Taiwan’s National Health Insurance (NHI) program including NHI enrollment files and medical service data (diagnoses, prescription drugs, and examinations). The NHI program is a compulsory single-payer healthcare system providing comprehensive healthcare for over 99% of the residents of Taiwan. The diagnostic data were coded using the International Classification of Diseases, Ninth Revision, Clinical Modification (ICD-9-CM) before 2015 and using the Tenth Revision (ICD-10-CM) after 2016.

### Ethics approval

This study was conducted in accordance with the Declaration of Helsinki^28^ and relevant institutional guidelines and regulations. The data used in the analysis was released by the HWDC. To protect the privacy of beneficiaries, scrambled and randomized identification numbers were assigned to ensure anonymity. Given the fully anonymized nature of the dataset, the requirement for informed consent was waived by the Research Ethics Committee of China Medical University & Hospital, Taichung, Taiwan (approval No. CMUH110-REC2-220). All study procedures were reviewed and approved by the Research Ethics Committee of China Medical University & Hospital.

### Study participants

The study population included 1,141,877 live births between 2010 and 2015, and followed up from birth to the end of 2020. We excluded infant using the following criteria: (i) infants with congenital anomaly (n=128,518); (ii) with intestinal obstruction (n=5,798); (iii) had been in intensive care unit (ICU) before 1-month (n=38,627); (iv) had been diagnosed with infantile colic but did not meet the criteria for diagnosis of the present study (n=52,860). After exclusions, this study included a total of 916,074 infants as the study population.

### Definition of infantile colic

Infantile colic cases were identified in the NHIRD using physician-recorded ICD-9-CM codes 789.0x and 780.9. A diagnosis required two or more outpatient visits or one hospitalization between the ages of one and five months. Code 789.0x was selected based on backward mapping from ICD-10 code R10.83 (infantile colic), and 780.9 was included to capture cases corresponding to R68.12 (excessive crying of infant). Infants were clinically diagnosed with infantile colic by pediatricians according to features consistent with the Rome III criteria and the Wessel’s rule—paroxysms of irritability, fussing, or crying lasting for ≥3 hours per day, occurring on ≥3 days per week, for at least one week, in an otherwise healthy infant. Because the study data were collected before 2015, the Rome IV criteria (published in 2016) were not applicable during the study period.

### Study design

#### Risk factors of incident colic

**Fig. 1:**
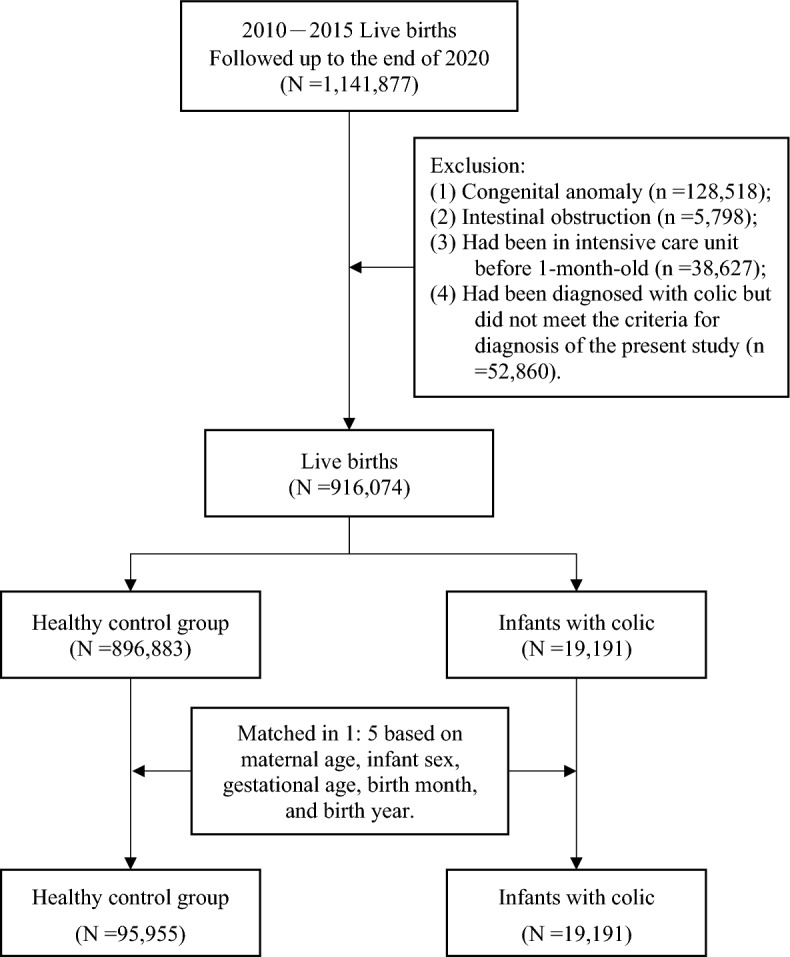
The process of study participant selection. The study included 1,141,877 infants born between 2010 and 2015, with follow-up from birth to the end of 2020. The infants were divided into two groups with a 1:5 matching for each infant with infantile colic, based on maternal age, infant sex, gestational age, birth month, and birth year.

We conducted a nested case-control study to analyze the risk factors of infantile colic. In order to control for confounders, we utilized the propensity score matching (PSM) method to achieve a 1:5 matching for each infant with infantile colic based on maternal age, infant sex, gestational age, birth month, and birth year. Following matching, the study included 19,191 infants with infantile colic in the case group and 95,955 healthy infants in the control group. Figure 1 illustrates the process of study participant selection.

The dependent variable was the infantile colic and the independent variable was the risk factors related to infantile colic including neonatal and maternal factors. The neonatal factors were accessed based on the medical records from birth and the ICD codes (Supplementary table 1), including birth body weight, large for gestational age (LGA), small for gestational age (SGA), cow’s milk protein allergy and antibiotics use before 1-month-old. The maternal factors included types of delivery, atopic diseases, post-partum depression, major depressive disorder, and irritable bowel syndrome (IBS), diagnosed within 1 year before giving birth.

#### The effect of infantile colic on long-term outcomes

We also conducted a cohort study to investigate the effect of infantile colic on long-term health outcomes in infants. The start date of observation was defined as the date when an infant received the first diagnosis of infantile colic. The same start date was applied to the PSM infants in the control group. All infants underwent two-year and five-year follow-ups, respectively. They were followed up from the start date until death, the occurrence of incident primary outcomes, or the end of the observation period, whichever occurred first. There were seven primary long-term outcomes of interest in the study: (i) functional constipation; (ii) functional diarrhea; (iii) IBS; (iv) other functional abdominal pain disorders; (v) autism spectrum disorder; (vi) attention deficit hyperactivity disorder (ADHD); (vii) atopic diseases including atopic dermatitis, allergic rhinitis, and asthma. All diagnoses were identified using ICD-9-CM codes before 2015 and ICD-10-CM codes after 2016, as summarized in Supplementary table 1. To enhance diagnostic accuracy, at least two outpatient visits or one hospital admission with relevant ICD codes were required. The confounders contained neonatal and maternal factors. The neonatal included gestational age, sex, birth body weight, cow milk protein allergy, LGA, SGA, and antibiotics use; the maternal factors included type of delivery, age, atopic disease, post-partum depression, major depressive disorder, and IBS.

### Statistical analysis

In terms of the risk factors of infantile colic, descriptive statistics such as numbers and percentage values were used to show distributions of the demographic characteristics, and the chi-square test was used to evaluate the difference in each risk factor between infants with and without colic. Additionally, the present study utilized conditional logistic regression to evaluate the associations between risk factors and infantile colic, and the results were presented as odds ratios (OR) with 95% confidence intervals (CI). The model was adjusted for maternal age, infant sex, gestational age, birth month, and birth year.

A Cox proportional hazards model (PH) was employed to evaluate the effect of infantile colic on long-term health outcomes. The censor was defined as the infant did not develop primary outcomes, lost to follow up, or died during the follow-up period. The results were reported as hazard ratios (HR) and 95% CI after adjusted for neonatal and maternal factors, including gestational age, sex, birth body weight, cow milk protein allergy, large for gestational age, small for gestational age, antibiotics use, type of delivery, maternal age, maternal atopic disease, post-partum depression, maternal major depressive disorder, and maternal irritable bowel syndrome.

All statistical analyses in this study were using SAS software version 9.4 (SAS Institute Inc., Cary, NC, USA). Statistical significance was defined as p < 0.05 in the study.

## Results

During 2010 to 2015, we enrolled a total of 19,191 infants with infantile colic. The incident rate of infantile colic was 2.09%. Table 1 shows the baseline characteristics between case and control group. Among the case group, 24.67% were born with a gestational age of ≤37 weeks, 51.75% were female, 7.78% had a birth body weight of <2,500g, and 1.18% had a birth body weight of >4,000g. Additionally, 80.52% of the cases were born to mothers aged 18-35 years, and 40.02% were born by cesarean section.

**Table 1:** Baseline characteristics.

Variables	Total		Control group		Case group		p-value
	N	%	N	%	N	%	
Total	115,146	100.00	95,955	100.00	19,191	100.00	
**Infant data**							
Gestational age (weeks)							1.000
≤37	28,410	24.67	23,675	24.67	4,735	24.67	
>37	86,736	75.33	72,280	75.33	14,456	75.33	
Sex							1.000
Female	59,586	51.75	49,655	51.75	9,931	51.75	
Male	55,560	48.25	46,300	48.25	9,260	48.25	
Birth body weight (g)							0.006
>4,000	1,499	1.30	1,273	1.33	226	1.18	
2,500-4,000	105,231	91.39	87,760	91.46	17,471	91.04	
<2,500	8,416	7.31	6,922	7.21	1,494	7.78	
**Maternal data**							
Type of delivery							<0.001
NSD	73,309	63.67	61,798	64.40	11,511	59.98	
Cesarean section	41,837	36.33	34,157	35.60	7,680	40.02	
Maternal age (years)							0.182
<18	703	0.61	604	0.63	99	0.52	
18-35	92,592	80.41	77,140	80.39	15,452	80.52	
>35	21,851	18.98	18,211	18.98	3,640	18.97	

NSD, normal spontaneous delivery.

### Maternal and infant risk factors of infantile colic—results from a case-control study

A total of 19,191 infants with colic and 95,955 matched controls were included in the case–control analysis. Infantile colic was significantly associated with several maternal and infant factors (Table 2). The percentage of infantile colic was 17.75% among infants with birth body weight <2,500g, and 17.92% among infants with antibiotics use. Infants who used antibiotics before 1 month old had an increased risk of infantile colic (OR =1.15, 95% CI =1.11–1.18) compared to those without antibiotics use in the adjusted model. The unadjusted model revealed that infants with a birth body weight <2,500g had a higher OR for infantile colic (OR = 1.03; 95% CI =1.03–1.09) compared to those with a normal birth body weight; however, this association was mitigated after adjusted for confounders. There were no statistically significant associations of infantile colic with LGA, SGA, or cow’s milk protein allergy.

**Table 2:** Odds ratios (95% confidence intervals) for infantile colic associated with maternal and infant risk factors.

Variables	Control	Infantile colic	Crude model		Adjusted model	
	Events (%)	Events (%)	OR (95% CI)	p-value	OR (95% CI)	p-value
**Infant factors**						
Birth body weight (g)						
>4,000	1,273 (84.92)	226 (15.08)	0.89 (0.77-1.03)	0.115	0.87 (0.75-1.01)	0.051
2,500-4,000	87,760 (83.4)	17,471 (16.60)	Reference		Reference	
<2,500	6,922 (82.25)	1,494 (17.75)	1.08 (1.02-1.15)	0.007	1.03 (0.97-1.09)	0.325
Cow’s milk protein allergy						
No	95,937 (83.34)	19,183 (16.66)	Reference		Reference	
Yes	18 (69.23)	8 (30.77)	2.24 (0.98-5.14)	0.058	2.06 (0.89-4.75)	0.090
Large for gestational age						
No	95,835 (83.33)	19,173 (16.67)	Reference		Reference	
Yes	120 (86.96)	18 (13.04)	0.75 (0.46-1.23)	0.255	0.75 (0.45-1.23)	0.255
Small for gestational age						
No	95,931 (83.33)	19,188 (16.67)	Reference		Reference	
Yes	24 (88.89)	3 (11.11)	0.63 (0.19-2.08)	0.444	0.57 (0.17-1.89)	0.357
Neonatal antibiotics use						
No	52,390 (84.4)	9,682 (15.60)	Reference		Reference	
Yes	43,565 (82.08)	9,509 (17.92)	1.18 (1.15-1.22)	<0.001	1.15 (1.11-1.18)	<.0001
**Maternal factors**						
Type of delivery						
NSD	61,798 (84.3)	1,1511 (15.70)	Reference		Reference	
Cesarean section	34,157 (81.64)	7,680 (18.36)	1.21 (1.17-1.25)	<0.001	1.17 (1.14-1.21)	<.0001
Atopic disease						
No	84,077 (83.89)	16,144 (16.11)	Reference		Reference	
Yes	11,878 (79.58)	3,047 (20.42)	1.34 (1.28-1.40)	<0.001	1.32 (1.27-1.38)	<.0001
Post-partum depression						
No	95,915 (83.34)	19,176 (16.66)	Reference		Reference	
Yes	40 (72.73)	15 (27.27)	1.88 (1.04-3.41)	0.037	1.60 (0.88-2.93)	0.126
Major depressive disorder						
No	95,694 (83.35)	19,115 (16.65)	Reference		Reference	
Yes	261 (77.45)	76 (22.55)	1.46 (1.13-1.88)	0.004	1.35 (1.04-1.76)	0.022
Irritable bowel syndrome						
No	95,390 (83.37)	19,034 (16.63)	Reference		Reference	
Yes	565 (78.25)	157 (21.75)	1.39 (1.17-1.66)	<0.001	1.35 (1.13-1.62)	0.001

NSD, normal spontaneous delivery; OR, odds ratio; CI, confidence interval.

In terms of maternal factors, the percentage of infantile colic 18.36% among infants with cesarean section; 20.42% among infants whose mothers had atopic disease; 22.55% among infants whose mothers had major depressive disorder; and 21.75% among infants whose mothers had IBS. The adjusted model revealed that infants delivered via cesarean section had a 1.17 times higher risk of infantile colic (95% CI =1.14–1.21), compared with normal spontaneous delivery. Infants whose mothers had atopic disease (OR =1.32, 95% CI =1.27–1.38), major depressive disorder (OR =1.35, 95% CI =1.04–1.76), and IBS (OR =1.35, 95% CI =1.13–1.62) had an increased risk of infantile colic (Table 2).

### The effect of infantile colic on long-term outcomes—the results from a cohort study

For the longitudinal cohort analysis, 19,191 infants with colic and 95,955 controls were followed for up to five years. Table 3 displays the effect of infantile colic to the long-term outcomes. In the two-year follow-ups, the incident rate per thousand person-years of every long-term outcome was higher in the case group than in the control group except autism spectrum disease. After controlling for neonatal and maternal factors, infants with colic had a higher risk of functional constipation (HR =2.17, 95 % CI =2.07–2.28), functional diarrhea (HR =1.45, 95 % CI =1.13–1.86), IBS (HR =1.76, 95 % CI =1.42–2.18), other functional abdominal pain disorders (HR =1.27, 95 % CI =1.22–1.32), ADHD (HR =3.15, 95 % CI =2.07–4.79), and atopic diseases (HR =1.17, 95 % CI =1.14–1.21).

**Table 3:** Hazard ratios (95% confidence intervals) for long-term outcomes associated with infantile colic during the two-year and five-year follow-up periods.

Variables	Control group		Case group		Case vs Control group (reference)			
	Events (%)	IR	Events (%)	IR	cHR	p-value	aHR*	p-value
**Two-year follow-up**								
Functional constipation	5,903 (6.15)	31.85	2,491 (12.98)	70.57	2.21 (2.11-2.31)	<0.001	2.17 (2.07-2.28)	<0.001
Functional diarrhea	271 (0.28)	1.41	80 (0.42)	2.09	1.48 (1.15-1.90)	0.002	1.45 (1.13-1.86)	0.004
Irritable bowel syndrome	319 (0.33)	1.67	114 (0.59)	2.98	1.79 (1.45-2.22)	<0.001	1.76 (1.42-2.18)	<0.001
Other functional abdominal pain disorders	12,870 (13.41)	73.21	3,157 (16.45)	94.68	1.29 (1.25-1.35)	<0.001	1.27 (1.22-1.32)	<0.001
Autism spectrum disorder	66 (0.07)	0.34	17 (0.09)	0.44	1.29 (0.76-2.20)	0.352	1.24 (0.73-2.12)	0.432
Attention deficit hyperactivity disorder	56 (0.06)	0.29	36 (0.19)	0.94	3.22 (2.12-4.89)	<0.001	3.15 (2.07-4.79)	<0.001
Atopic diseases	19,155 (19.96)	112.66	4,471 (23.30)	135.03	1.20 (1.16-1.24)	<0.001	1.17 (1.14-1.21)	<0.001
**Five-year follow-up**								
Functional constipation	8,719 (9.09)	46.29	3,329 (17.35)	92.11	1.78 (1.71-1.86)	<0.001	1.77 (1.70-1.84)	<0.001
Functional diarrhea	349 (0.36)	1.82	96 (0.50)	2.51	1.48 (1.18-1.86)	<0.001	1.47 (1.17-1.85)	<0.001
Irritable bowel syndrome	437 (0.46)	2.28	137 (0.71)	3.58	1.70 (1.41-2.07)	<0.001	1.69 (1.40-2.06)	<0.001
Other functional abdominal pain disorders	19,517 (20.34)	106.07	3,858 (20.10)	112.78	1.23 (1.19-1.28)	<0.001	1.22 (1.18-1.26)	<0.001
Autism spectrum disorder	354 (0.37)	1.84	93 (0.48)	2.42	1.10 (0.87-1.38)	0.416	1.06 (0.84-1.33)	0.647
Attention deficit hyperactivity disorder	952 (0.99)	4.92	236 (1.23)	6.10	1.19 (1.03-1.38)	0.016	1.20 (1.04-1.39)	0.015
Atopic diseases	32,357 (33.72)	174.45	7,328 (38.18)	201.24	1.12 (1.09-1.15)	<0.001	1.11 (1.08-1.14)	<0.001

IR, incident rate of per thousand person-year; cHR, crude hazard ratio; aHR, adjusted hazard ratio.

^*^ Analysis adjustment variables contained neonatal factors (gestational age, sex, birth body weight, cow milk protein allergy, large for gestational age, small for gestational age, and antibiotics use) and maternal factors (type of delivery, age, atopic disease, post-partum depression, major depressive disorder, and irritable bowel syndrome).

Similar results were observed in the five-year follow-up analysis. Compared with the control group, infants with infantile colic had a higher risk of functional constipation (HR =1.77, 95 % CI =1.70–1.84), functional diarrhea (HR =1.47, 95 % CI =1.17–1.85), IBS (HR =1.69, 95 % CI =1.40–2.06), other functional abdominal pain disorders (HR =1.22, 95 % CI =1.18–1.26), ADHD (HR =1.20, 95 % CI =1.04–1.39), and atopic diseases (HR =1.11, 95 % CI =1.08–1.14) (Table 3).

## Discussion

Our large nationwide population-based study identified risk factors of infantile colic, including neonatal antibiotic use, as well as maternal factors including cesarean section, maternal atopic diseases, major depressive disorder and IBS. Moreover, our investigation revealed associations between infantile colic and long-term health implications, such as DGBI, ADHD, and atopic diseases. These findings suggest the multifaceted nature of infantile colic and its potential pathophysiology and long-term health outcomes, emphasizing the importance of early recognition and targeted intervention strategies.

Although previous studies have reported the prevalence of infantile colic to be between 1.9% and 19.2%(1), our study identified a lower prevalence of 2.09%. This discrepancy may be partly explained by the use of stricter inclusion criteria; we included only infants with either two or more outpatient visits or one hospital admission coded with colic-related ICD-9-CM codes between 1 and 5 months of age. When the case definition was broadened to include infants with a single outpatient diagnosis, the prevalence increased to 5.57%, suggesting that coding thresholds substantially influence case identification. In addition, infants admitted to the NICU within the first month of life were excluded, which may have further contributed to the lower prevalence estimate. Lastly, ICD code underreporting is also possible, as not all clinicians consistently assign specific diagnostic codes for colic during outpatient encounters, particularly when symptoms are mild or self-limiting.

A few studies have reported the risk factors of infantile colic in newborns. Preterm delivery and SGA are associated with an increased risk of infantile colic^29–31^. In our study, gestational age was matched in both groups, and we excluded infants admitted to the ICU after birth. Because the premature infants often have some comorbidities including heart disease, brain disease, and increased susceptibility to regurgitation, their symptoms can closely resemble those of infantile colic. In this study, we analyzed the effect of birth body weight on the infants. However, SGA did not show statical significance in either the unadjusted or adjusted model. Low birth weight (<2500 gm) was positively associated with infantile colic compared to those with a birth body weight of 2,500-4,000g in the unadjusted model; however, the association was mitigated after adjusted for confounders. Additionally, we found that infants with antibiotics use before 1-month-old had an increased risk of infantile colic compared to those without antibiotics use. Early-life antibiotic use can induce immune dysregulation, disrupt barrier functions, alter microbiota composition, and affect gut sensorimotor functions, potentially contributing to the development of infantile colic^5,32^. This result is consistent with the limited but promising evidence supporting the beneficial effects of probiotics in treating infantile colic among full-term infants^33–35^.

In terms of maternal risk factors, we observed that infants born through cesarean section and those whose mothers had atopic diseases, major depressive disorder, and IBS had an elevated risk of infantile colic. Several studies have shown that cesarean section can alter the gut microbiota of infants by preventing transmission of maternal-derived pioneer gut bacteria at birth^32,36,37^. Cesarean sections and early-life antibiotic use may lead to similar outcomes in infants, reducing microbiota diversity and delaying microbiota maturation. However, dysbiosis may not be the sole reason for the development of infantile colic; other factors may also contribute to its occurrence. Maternal psychological issues, atopic diseases^38,39^ or IBS also play a role in the development of infantile colic in their offspring. Prospective studies have found that maternal psychiatric problems, including anxiety, major depression, and postpartum depression, are associated with infantile colic^40–42^. It is believed that a healthy interaction between mother and infant is associated with the safe short- and long-term mental and physical development of the offspring. However, infants whose mothers had post-partum depression did not have an increased risk of infantile colic (OR = 1.60, 95% CI = 0.88-2.93, P = 0.126) in the adjusted model in our study. The relationship may be influenced by whether mothers seek medical help or by their self-awareness of postpartum depression. In a French study, early clinical signs in infants, including sleep difficulties, feeding problems, and inconsolable crying, can alert the medical team to potential psychological suffering in the mother^43^. It is important that when treating infants with infantile colic, pediatricians also refer the mothers to a psychiatrist if needed.

We also found relationships between infantile colic and several long-term outcomes using a cohort study design. The results were similar in both the 2-year and 5-year follow-ups. Infants with colic had a higher risk of functional constipation, functional diarrhea, IBS, other functional abdominal pain disorders, ADHD, and atopic diseases. Previous studies have shown that infantile colic is associated with childhood DGBI and atopic diseases^23,24,26,44,45^. The results of these studies suggested that infantile colic may be an early manifestation of these disorders or shares the similar pathophysiology. Altered microbiota and trans-mucosal passage of antigens across the immature intestinal barrier, which affect immune system development, may play a crucial role in the pathogenesis of DGBI and atopic diseases^9,10^. However, there is currently a lack of large prospective studies addressing the treatment of infantile colic and the prevention of long-term disorders. We are also interested in brain development and behavioral diseases in children and found that infantile colic is associated with ADHD but not with autism spectrum disorder. Prospective studies have shown that a significantly higher percentage of preschool children with a history of infantile colic exhibit hyperactivity, mood problems, and overall behavior problems^46,47^. The importance of the brain-gut axis should be emphasized in infantile colic; however, we cannot exclude other pre-existing factors such as genetic and environmental influences in our study.

This study has several limitations. First, the TMCHD lacks detailed clinical and behavioral information. Symptom-level characteristics of infantile colic—such as the age at first presentation, duration and timing of crying, and associated sleep patterns—were unavailable, as the database contains administrative claims rather than detailed clinical records. Likewise, data on certain potential risk factors, including birth order, parental consanguinity, and specific feeding practices (e.g., exclusive breastfeeding or infant formula), were not recorded. Information on treatments such as *L. reuteri* supplementation was also unavailable because this probiotic is self-paid and not covered by the National Health Insurance system. Future prospective cohort studies are warranted to explore these potential risk factors and their relationships with infantile colic. Second, unmeasured parental factors may have influenced the observed associations. Parents with higher anxiety or health concerns may seek medical care more frequently for their children, leading to potential detection bias and overestimation of the associations. Future studies incorporating parental psychosocial variables or total medical encounter data would help further address this residual confounding. Third, the follow-up period in our study was limited to five years. Some conditions, such as irritable bowel syndrome and migraine, may manifest more prominently during adolescence, while attention-deficit/hyperactivity disorder may be diagnosed after the age of five. Extending the follow-up period beyond five years could provide a more comprehensive understanding of the long-term impacts of infantile colic.

## Conclusions

In conclusion, our nationwide population-based study provides valuable insights into the potential risk factors and long-term health implications of infantile colic, highlighting the need for further prospective studies to elucidate the definite pathophysiology and develop effective interventions. Dysbiosis may play an important role in infantile colic, but other genetic and environmental factors should be taken into consideration. It also underscores the importance of a holistic approach in pediatric care, including the consideration of maternal psychological health, to ensure comprehensive care for both the infant and the mother.

## Supplementary Information

Supplementary Information.

## Data Availability

The data that support the findings of this study are available from the Health and Welfare Data Science Center (HWDC), Ministry of Health and Welfare, Taiwan. However, restrictions apply to the availability of these data, which were used under license for the current study and are therefore not publicly available. Data are available from the authors upon reasonable request and with permission of the HWDC.

## Abbreviations

DGBI: Disorders of gut-brain interaction
ICU: Intensive care unit
ICD: International classification of diseases
IBS: Irritable bowel syndrome
ADHD: Attention deficit hyperactivity disorder
NSD: Normal spontaneous delivery
LGA: Large for gestational age
SGA: Small for gestational age
OR: Odds ratio
HR: Hazard ratio
CI: Confidence intervals
PSM: Propensity score matching
