# Supplementary material for: Risk factors and long-term outcomes of infantile colic: A nationwide population-based study
Source: Sci Rep. 2026 Jan 13;16:4567. doi: 10.1038/s41598-025-34646-4 (PMC12868711; doi:10.1038/s41598-025-34646-4)
Supplement: Supplementary file 1 — Supplementary Information. [file 41598_2025_34646_MOESM1_ESM.docx]

**Supplemental table 1.**

The international classification of diseases (ICD) codes used in this study are summarized in Supplemental table 1. The diagnostic data were coded using ICD-9-CM before 2015 and ICD-10-CM after 2016.

| Diagnosis | ICD codes |
| --- | --- |
| Infantile colic | ICD-9-CM 789.0x, 780.9 |
| Large for gestational age | ICD-9-CM 766.1 |
| Small for gestational age | ICD-9-CM 764.1 |
| Cow milk protein allergy | ICD-9-CM V15.02 |
| Post-partum depression | ICD-9-CM 648.4 |
| Major depressive disorder | ICD-9-CM 296.2, 296.3 |
| Irritable bowel syndrome | ICD-9-CM 564.1  ICD-10-CM K58 |
| Functional constipation | ICD-9-CM 564.0  ICD-10-CM K59.00-K59.02, K59.09 |
| Functional diarrhea | ICD-9-CM 564.5  ICD-10-CM K59.1 |
| Other functional abdominal pain disorders | ICD-9-CM 346.20, 346.21, 536.8, 564.89, 564.9, 789.00  ICD-10-CM G43.A0, G43.A1, G43.D0, G43.D1, K30, K59.8, K59.9, R10.9 |
| Autism spectrum disorder | ICD-9-CM 299.0, 299.8  ICD-10-CM F84.0, F84.5 |
| Attention deficit hyperactivity disorder | ICD-9-CM 314  ICD-10-CM F90, F98.8 |
| Atopic dermatitis | ICD-9-CM 477  ICD-10-CM J30 |
| Allergic rhinitis | ICD-9-CM 691.8  ICD-10-CM L20 |
| Asthma | ICD-9-CM 493  ICD-10-CM J45 |
